# 

**DOI:** 10.1192/bjb.2019.73

**Published:** 2020-04

**Authors:** Adam G. Van Hagen

**Affiliations:** A learning designer and education writer at Hubble Studios, Cape Town, South Africa. Email: adam@hubblestudios.com

As interest in psychedelic research continues to increase, it is clear that a new conceptual framework is needed to investigate phenomena associated with altered states of consciousness. While research has focused primarily on their psychotherapeutic and entheogenic uses, few studies have dared to consider psychedelics as potent tools for enhancing cognition, conducting conceptual research and improving complex problem-solving. Sensing an opportunity to kick-start an intellectual revolution, Roberts introduces ‘multistate theory’ as a potential framework to guide new exploration of altered states of consciousness.

A core tenant of multistate theory is the rejection of the ‘singlestate fallacy’, which Roberts defines as the erroneous assumption that all worthwhile skills, abilities and knowledge reside in our default waking state. He argues that our default state is simply one of many possible states of consciousness (or ‘mindbody states’) that the mind can produce and operationalise. Directly criticising mainstream science and philosophy's understanding of intelligence and consciousness, Roberts provides ample evidence that cognitive processes qualifying as intelligence reside in other mindbody states.

*MindApps* explores a simple analogy – as apps are to smart devices, ‘mindapps’ are to the brain–mind complex. Therefore, any agent of psychological change (both drug and non-drug) that produces a mindbody state can be considered a mindapp. Different kinds of mindapp, of which psychedelics are perhaps the most potent, ultimately produce disparate mindbody states. Consequently, Roberts challenges the reader to consider a new age of ‘mind design’, where mindapps are used in combinations to investigate mindbody states, create new ones and uncover the full extent of the human mind. As transhumanist and transpersonal perspectives begin to intersect, could it be possible to design minds that far surpass the functions and capabilities of our current ones?

Multistate theory is a novel conceptual framework that sits at the crossroads of science, philosophy, spirituality, humanities and the arts. Considering the evidence illustrating the efficacy of psychedelics and other mindapps as catalysts for substantive psychological change, multistate theory can serve as a guide not only for systematically investigating mindapps and the cognitive processes that characterise mindbody states, but also for conducting conceptual research.

While he is clearly optimistic about the future, Roberts is quick to highlight that *MindApps* is merely the start of a new conversation about what it means to have a mind. However, given his contributions to psychedelic research and education over the past 30 years, Roberts's conclusions are deserving of a wider audience. As we shift from the age of information to the age of experience, his clarion call for bold innovation should be heard by all who wish to pioneer the development of new research questions, creative methodologies and engaging education for future generations of mind-designers.

